# Differential Expression of miR-145 in Children with Kawasaki Disease

**DOI:** 10.1371/journal.pone.0058159

**Published:** 2013-03-06

**Authors:** Chisato Shimizu, Jihoon Kim, Petra Stepanowsky, Christine Trinh, Hubert D. Lau, Johnny C. Akers, Clark Chen, John T. Kanegaye, Adriana Tremoulet, Lucila Ohno-Machado, Jane C. Burns

**Affiliations:** 1 Department of Pediatrics, University of California San Diego, La Jolla, California, United States of America; 2 Division of Biomedical Informatics, Department of Medicine, University of California San Diego, La Jolla, California, United States of America; 3 Department of Neurosurgery, University of California San Diego, La Jolla, California, United States of America; 4 Rady Children’s Hospital San Diego, San Diego, California, United States of America; The Ohio State University, United States of America

## Abstract

**Background:**

Kawasaki disease is an acute, self-limited vasculitis of childhood that can result in structural damage to the coronary arteries. Previous studies have implicated the TGF-β pathway in disease pathogenesis and generation of myofibroblasts in the arterial wall. microRNAs are small non-coding RNAs that modulate gene expression at the post-transcriptional level and can be transported between cells in extracellular vesicles. To understand the role that microRNAs play in modifying gene expression in Kawasaki disease, we studied microRNAs from whole blood during the acute and convalescent stages of the illness.

**Methodology/Principal Findings:**

RNA isolated from the matched whole blood of 12 patients with acute and convalescent Kawasaki disease were analyzed by sequencing of small RNA. This analysis revealed six microRNAs (miRs-143, -199b-5p, -618, -223, -145 and -145* (complementary strand)) whose levels were significantly elevated during the acute phase of Kawasaki disease. The result was validated using targeted qRT-PCR using an independent cohort (n = 16). miR-145, which plays a critical role in the differentiation of neutrophils and vascular smooth muscle cells, was expressed at high levels in blood samples from acute Kawasaki disease but not adenovirus-infected control patients (*p* = 0.005). miR-145 was also detected in small extracellular vesicles isolated from acute Kawasaki disease plasma samples. Pathway analysis of the predicted targets of the 6 differentially expressed microRNAs identified the TGF-β pathway as the top pathway regulated by microRNAs in Kawasaki disease.

**Conclusion:**

Sequencing of small RNA species allowed discovery of microRNAs that may participate in Kawasaki disease pathogenesis. miR-145 may participate, along with other differentially expressed microRNAs, in regulating expression of genes in the TGF-β pathway during the acute illness. If the predicted target genes are confirmed, our findings suggest a model of Kawasaki disease pathogenesis whereby miR-145 modulates TGF-β signaling in the arterial wall.

## Introduction

The self-limited nature of the inflammation in Kawasaki disease (KD) is unique among vasculitis syndromes and argues for effective host mechanisms that successfully down regulate inflammation to terminate the acute, febrile phase of the illness. Despite cessation of fever and other signs of inflammation, 25% of untreated children will develop permanent damage to the coronary arterial wall that results in aneurysm formation [1], [2]. The current paradigm is that genetically susceptible children are exposed to an unknown trigger that elicits an immune response directed against some components of the arterial wall [3]. Key genetic pathways implicated in disease susceptibility include calcium signaling pathways and the transforming growth factor (TGF)-β pathway [4]–[7]. Recent studies of the histopathology of aneurysmal coronary arteries from KD autopsies suggest that myofibroblasts play an important role in KD pathogenesis by secreting pro-inflammatory cytokines and recruiting inflammatory cells to the arterial wall [8], [9]. Immunohistochemical studies implicate signaling through the TGF-β pathway as a possible mechanism of endothelial- or epithelial-to-mesenchymal transformation resulting in myofibroblasts infiltrating the vessel wall [9].

We postulated that the TGF-β signaling during the acute vasculitis may be modulated through inter-cellular transport of microRNAs (miRs) [10]. miRs are small non-coding RNAs that control gene expression either by inducing transcript degradation or by blocking translation. miRs that influence expression of genes associated with immune regulation [11] and cardiovascular remodeling [12] have been described. Transport of miRs between the endothelial cells and smooth muscle cells has also been reported [13]. The regulation of gene expression at the miR level has not been previously investigated in KD. Therefore, we profiled differentially expressed miRs in the blood of acute and convalescent KD patients and adenovirus-infected control patients to define the miR-ome and assess the potential role that these miRs might play in modulating gene expression. We focused on one differentially expressed miR, miR-145, because of its role in vascular biology and demonstrated its transport in extracellular vesicles.

## Materials and Methods

### Subjects

The demographic and clinical characteristics of study subjects are presented in Table 1 and Table S1. All patients diagnosed with KD had fever for at least three days and met at least four of five clinical criteria for KD (rash, conjunctival injection, cervical lymphadenopathy, oral mucosal changes, and changes in the extremities) or three of five criteria and coronary artery abnormalities documented by echocardiogram [14]. Patients with adenovirus infection, which mimics many of the clinical and laboratory findings in acute KD, were prospectively enrolled as febrile control subjects and the diagnosis of adenovirus infection was subsequently made by a positive direct or indirect fluorescent antibody test on nasopharyngeal cells or by viral culture of secretions on nasopharyngeal or throat swabs. Complete blood counts were performed on the same sample used for miR analysis during the acute phase and prior to IVIG administration for the KD subjects. Coronary artery dimensions were described by the variable Z_worst_, which was defined as the maximal Z score (standard deviation units from the mean) of the internal diameter of the left anterior descending and right coronary arteries normalized for body surface area, within the first year after KD onset.

**Table 1:** Demographic and clinical characteristics of subjects.

		Sequencing	RT-PCR	RT-PCR	AdditionalRT-PCR
		Whole blood	Whole blood	Extracellular vesicles	Whole blood
Kawasaki disease	Number of subjects	12	16	14	8
	Male, No.(%)	8 (67)	9 (56)	5 (36)	5 (62.5)
	Age in months, median (range)	35.6 (10–80)	39.5 (11–75)	39.5 (4.4–131)	19.6 (7.8–38.1)
	Illness day of sample collection, median (range)				
	Acute	7 (3–10)	6 (3–11)	6 (3–12)	6.5 (6–12)
	Convalescent	41 (37–462)	45 (27–443)	ND	45.5 (41–380)
	Ethnicity, No. (%)				
	Hispanic	6 (50)	3 (19)	6 (43)	2 (25)
	Asian	2 (17)	4 (25)	2 (14)	2 (25)
	Caucasian	1 ( 8)	2 (12)	1 ( 7)	1 (12.5)
	African American	1 ( 8)	2 (12)	0 ( 0)	1 (12.5)
	More than one race	2 (17)	3 (19)	5 (36)	2 (25)
	Unknown	0 ( 0)	2 (12)	0 ( 0)	0 ( 0)
	CA status No. (%)				
	Normal	9 (75)	9 (56)	11 (79)	4 (50)
	Dilated	2 (17)	5 (31)	3 (21)	0 (0)
	Aneurysm	1 ( 8)	2 (13)	1 ( 7)	4 (50)
	Treatment responder†	12 (100)†	16 (100)†	10 (71)	7 (87)
Adenovirus infection	Number of subjects	6	14	ND	ND
	Male, No. (%)	4 (67)	9 (64)		
	Age in months, median (range)	54.8 (11.5–84.2)	45.3 (13.3–105.5)		
	Illness day of sample collection, median (range)	6 (2–10)	6 (4–7)		
	Ethnicity, No. (%)				
	Hispanic	2 (33)	3 (21)		
	Asian	0 ( 0)	0 ( 0)		
	Caucasian	2 (33)	3 (21)		
	African American	0 ( 0)	0 ( 0)		
	More than one race	1 (17)	4 (29)		
	Unknown	1 (17)	4 (29)		

† Fever resolved after single infusion of IVIG, CA: coronary artery, ND.: not done.

### Sample Collection and RNA Extraction

For whole blood RNA, 2.5 mL of blood were collected in PAXgene® tubes (PreAnalytiX, Hombrechtikon, Switzerland) from KD subjects (n = 12 for sequencing, n = 16 for quantitative reverse transcription polymerase chain reaction (q*RT*-*PCR*)) at the time of diagnosis prior to IVIG treatment (acute phase) and again approximately 1 month to 1 year after the resolution of the acute illness when the erythrocyte sedimentation rate, platelet count, and C-reactive protein levels had returned to normal (convalescent phase). An additional four KD subjects with coronary artery aneurysms (Z_worst_>4.5) and four subjects with normal coronary artery internal dimension by echocardiography were analyzed for miR-145 transcript abundance during the acute, subacute (illness day 19–24), and convalescent phases. The estimated power for 12 subjects was 86% using Wilcoxon signed-rank test with matched-pairs assuming effect size 1 and significance level 0.05. For adenovirus-infected control subjects (n = 6 for sequencing, n = 14 for qRT-PCR), blood was collected only at the acute stage. PAXgene tubes were frozen at −20°C until RNA extraction. For extracellular vesicle isolation from KD subjects in the acute phase (n = 14), blood was collected in tubes containing sodium citrate. All plasma samples were centrifuged within 48 hours after sample collection and frozen at −70°C until use. RNA was isolated using the PAXgene Blood miRNA Kit (PreAnalytiX, Hombrechtikon, Switzerland) that enriches for small RNA species according to the manufacturer’s protocol. RNA was extracted from extracellular vesicles using miRNeasy mini kit (Quiagen, Valencia, CA) according to manufacturer’s protocol.

### Deep Sequencing

A small RNA library (<30 nucleotide (nt)) was prepared from 1 µg of total RNA using the small RNA sample preparation kit (Cat. FC-930-1004, Illumina) following the manufacturer’s protocol. Briefly, following linker ligation and RT-PCR amplification, the bands corresponding to small RNAs ligated with linkers between 75-125bp were excised from a 5% *Tris/Borate/EDTA (*TBE) *polyacrylamide gel* electrophoresis **(** *PAGE* **)** gel (Biorad). Cluster generation and sequencing on Illumina HiSeq 2000 was performed at the Biomedical Genomics laboratory (BIOGEM) (http://microarrays.ucsd.edu/).

### qRT-PCR

To validate differentially expressed miRs, qRT-PCR was performed using commercially available stem-loop qRT-PCR primers (Life Technologies, Grand Island, NY) for miR-23a (Assay ID 000399), miR-145 (Assay ID 002278), miR-199b-5p (Assay ID 000580), miR-223 (Assay ID 002295), miR-618 (Assay ID 001593), and miR-1271 (Assay ID 002779). For miRs -96, -100, -125b, -143, -145* (complementary strand of miR-145), and -501-3p with dominant read sequences that differed from miRBase annotated sequences (used to design commercially available primers), we designed custom stem-loop RT-primers to match the dominant read sequences using commercially available design tools (Life Technologies, Grand Island, NY). qRT-PCR was performed according to manufacturer’s instructions with Super Script III reverse transcriptase (Life Technologies, Grand Island, NY). Relative abundance of the target transcripts from whole blood was normalized to the expression level of small nucleolar RNA, C/D box 48 (RNU48) (Assay ID 001006) according to the manufacturer’s instructions.

### Isolation and Characterization of Circulating Extracellular Vesicles

Frozen plasma samples (1–5 mL) from acute KD subjects were thawed, diluted 1∶1 with PBS, and centrifuged for 15 min at 2,000 g (Sorvall Legend RT) at 4°C to remove any cellular debris. Supernatants were transferred to 5 mL ultracentrifuge tubes and the volume brought up to 5 mL with additional PBS. Samples were centrifuged (Beckman rotor Ti45) for 1 hour at 20,000 g at 4°C to remove larger vesicles in the pellet. The supernatant was then transferred to a new tube and centrifuged for 2 hours at 120,000 g at 4°C to collect small vesicles in the pellet. The pellet was resuspended in PBS and subjected to a second round of centrifugation for 70 min at 120,000 g at 4°C. The supernatant was decanted and the pellet was resuspended overnight at 4°C in 20–60 µL of sterile PBS. For determination of size and number of vesicles based on Brownian motion, the NanoSight (NanoSight LM10 HS, Amesbury, UK) equipped with a 532 nm laser was used. Resuspended vesicles were diluted 1∶25 to 1∶100 with sterile PBS and applied to the NanoSight which calculated particle size and number.

### miRs in Extracellular Vesicles

To test the possibility that miRs are transported in extracellular vesicles, vesicles isolated by ultracentrifugation were incubated (37°C, 30 minutes) with 2 µg/mL protease-free RNase A (Sigma-Aldrich, St. Louis, MO) and aliquots were subjected to the following conditions before RNA extraction using miRNeasy: a) 500 µg/mL proteinase K (Sigma-Aldrich, St. Louis, MO) dissolved in RNAse-free water at 37°C for 60 minutes, followed by heat inactivation of the protease at 60°C for 10 minutes, b) 0.1% Triton-X at 37°C for 60 minutes, and c) condition a) followed by condition b). Controls were aliquots treated only with or without RNase A treatment. miR-145 transcript levels were measured by qRT-PCR using primers described above.

### Preprocessing and Alignment

After removing the 3′ adapter sequence, the trimmed read was discarded if its length was shorter than 17 nt or longer than 27 nt. The reads containing more than two mismatches were also removed. The remaining reads were mapped to biological reference databases with Bowtie2 [15] allowing no mismatch to human genome (hg19 build), miR precursor (other than mature or star sequences) (miRBase build 18) [16] and non-coding RNA (ncRNA) (fRNAdb version 3.4) [17]. For known miRs, genome-mapped reads were used as long as they did not match to any of the known non-coding RNAs. For novel miRs, genome-mapped reads that did not map to any ncRNA were quantified and used for construction of a probabilistic model through miRDeep2 [18].

### Differential Expression

Differential expression analysis was performed using the R package DESeq, which includes a negative binomial-based statistical test shown to correctly estimate the effects of biological variability in small sample sizes [19]. Fold-change was calculated on the normalized read count through a variance-stabilizing transformation. For decision-making about the significance of differential expression, adjusted *p*-values were used to correct for multiple hypotheses, following the Benjamini-Hochberg procedure [20].

### Pathway Enrichment

The six validated miRs from among the top ten differentially expressed miRs were subjected to pathway enrichment analysis. A union set of target genes for the six miRs was derived from DIANA-microT-CDS [21], a prebuilt miR-target database with a threshold for the miR target gene (miTG) score of 0.7. The score is a weighted sum of a) the miR Recognition Element (MRE) score that is assessed according to binding behavior, which includes free energy calculations, between the miR driver sequences in the 5′ and 3′UTR sequences of the target gene and b) the degree of conservation in other species.

Next, a subset of this union set overlapping with Kyoto Encyclopedia of Genes and Genomes (KEGG) pathways was derived. The minimum number of genes per pathway was set at 10 for filtering purposes. A one-sided Fisher’s exact test was performed for each KEGG pathway and the corresponding *p*-value was calculated. DIANA-miRPath (version 2.0, http://microrna.gr/miRPathv2) [22] and Python scripts were used for analysis.

### Statistical Analyses

Spearman correlations were calculated between levels of different miRs and between miR levels and values from clinical laboratory testing performed on the same blood samples. Correlations were sought between acute miR levels and Z*_worst_*.

### Ethics Statement

All patients were enrolled at Rady Children’s Hospital San Diego after obtaining written parental informed consent and patient assent as appropriate. The study protocol was reviewed and approved by the University of California–San Diego Institutional Review Board.

## Results

### Small RNA Sequencing

**Figure 1:**
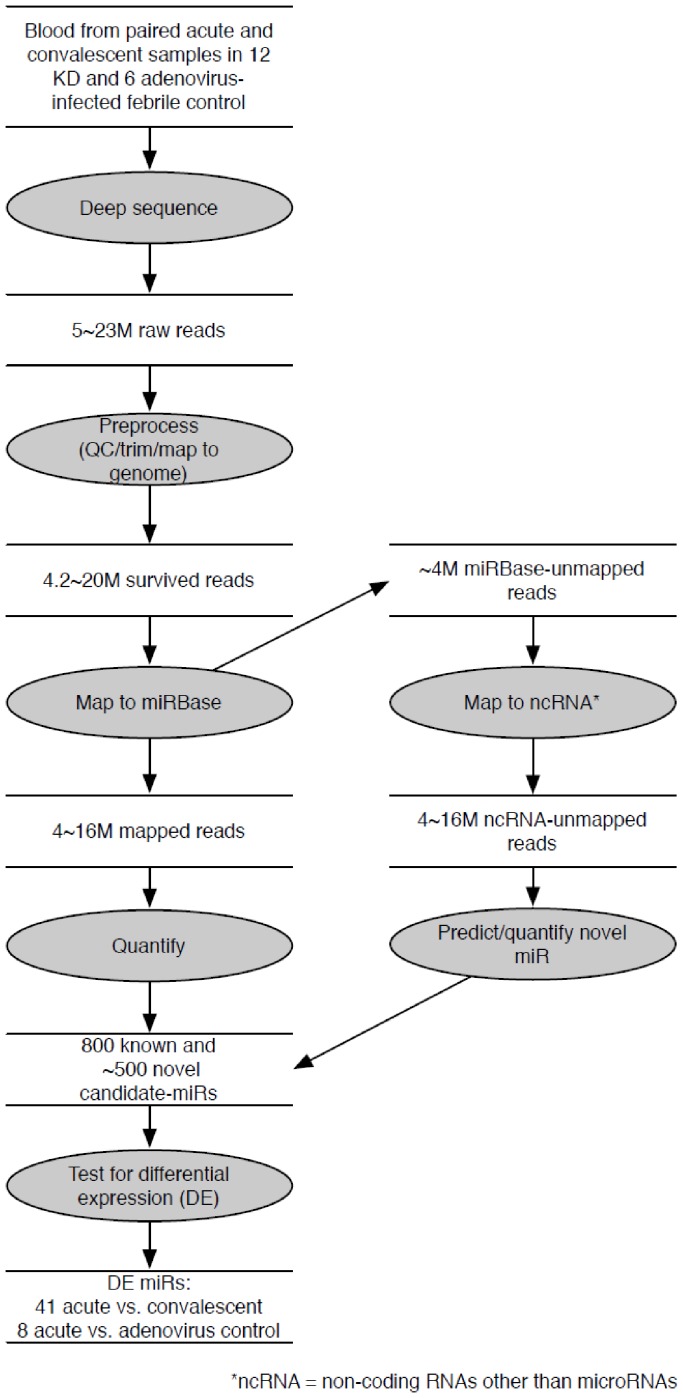
Flowchart of analytic strategy used to annotate known miRs and find differentially expressed miRs. Total RNA extracted from whole blood from subjects with KD and acute adenovirus infection was sequenced using Illumina HighSeq. A custom-designed in-house tool identified known and novel miRs from among the small non-coding RNA species and analyzed differential expression of known miRs between acute vs. convalescent KD samples and acute KD vs. adenovirus-infected samples.

We sequenced the 30 libraries (acute and convalescent KD samples, n = 12 paired samples, and acute adenovirus-infected controls, n = 6), each generating 5–23 million raw reads (Figure 1). We deposited the raw sequencing data in the NCBIGene Expression Omnibus (http://www.ncbi.nlm.nih.gov/geo, series accession number GSE pending). After removing low quality reads such as unread/no adaptor sequence (4.6–6.1% of total reads), reads shorter than 17 nt (0.4–10.2% of total reads), and reads longer than 27 nt (4.9–8.9 % of total reads) by enforcing Phred quality score of 10 and above, 79–89 % (median 86% of total reads) were selected as reads for further analysis. Of these qualified reads, 3–16 million reads (79–94% of total reads) matched to the human genome (build hg19) and were used for small non-coding RNA analysis. Of those sequence reads, 3–17 million reads (66–82 % of total reads, 98–99% of human genome-matched reads) were identified as known miRs annotated in miRBase (build 18) with a perfect match of the seed sequences in the 3′ end. The most abundant miR species were 22 nt in length, as expected.

Other small RNA sequences were also detected by mapping to databases of lincRNA, rRNA, snRNA, snoRNA and tRNA (Table S2). Genome-mapped reads that were not mapped to any ncRNA were used to predict novel miRs. We reasoned that miRs that are differentially expressed during the acute and the convalescent stages of the disease should be important in disease pathogenesis. None of the putative novel miRs were differentially expressed, so we focused on known miRs whose expression pattern changed over the course of the illness.

### Differentially Expressed miRs in Acute vs. Convalescent KD

To define a miR profile associated with acute KD, we compared acute vs. convalescent miR transcript abundance patterns. We found that 697 miRs and 672 miRs were expressed with at least 1 read in at least 1 sample from acute or convalescent KD subjects (n = 12), respectively, and 41 known miRs (registered in miRBase) were differentially expressed between acute and convalescent samples (Table S3) (p = 4.6×10^−7^–4.3×10^−2^). All 41 miR transcript levels were higher in the acute samples. The top 10 differentially expressed miRs included three miRs (miR-143, -145, -145*) from the miR-143/145 cluster on chromosome 5q32 (Table 2). Analysis of read alignments to known miR precursors from the miRBase revealed many isomiRs [23] (Figure S1). For miR-143, we found 7–18 unique reads per acute KD sample (median 11) and 3–14 unique reads per convalescent KD sample (median 5).The dominant read for miR-143 was 1 nt shorter than the miRBase annotated sequence. For miR-145, the dominant read was identical to the miRBase annotated sequence and we found 5–16 unique reads per acute KD sample (median 12) and 0–9 unique reads per convalescent KD sample (median 4).

**Table 2:** Top 10 differentially expressed miRs: Acute vs. Convalescent KD.

	Expression levels†			
miR	Acute	Convalescent	Fold change	*p*-value‡
miR-143	1.42	0.20	7.1	4.67E-07
miR-199b-5p	0.077	0.010	7.8	2.10E-06
miR-618	0.021	0.003	7.7	1.20E-04
miR-223	4.47	1.20	3.7	2.82E-04
miR-145	0.067	0.016	4.1	4.18E-04
miR-96	0.46	0.17	2.7	5.45E-04
miR-23a	0.53	0.14	3.7	1.26E-03
miR-582-3p	0.037	0.007	5.1	1.34E-03
miR-145*	0.007	0.001	5.7	1.54E-03
miR-221*	0.023	0.006	3.6	3.90E-03

† read counts normalized by total reads per sample.

‡ corrected for multiple sample comparison.

* complementary sequence (Star).

**Figure 2:**
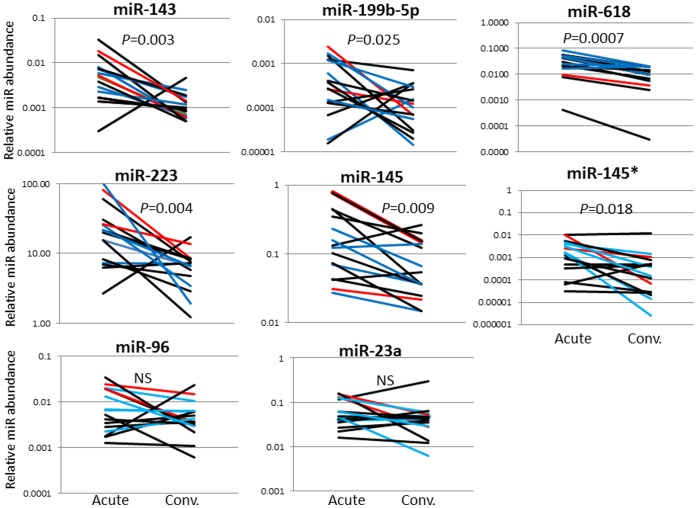
qRT-PCR validation of miRs differentially expressed in acute and convalescent KD whole blood samples. qRT-PCR confirmed differential expression between acute and convalescent blood samples for six of eight miRs. Red line: subjects with coronary artery aneurysm, Blue line: subjects with transiently dilated coronary arteries, Black line: subjects with normal coronary arteries. Comparisons by the Wilcoxon signed rank test. *denotes complementary sequence. NS = not significant.

**Figure 3:**
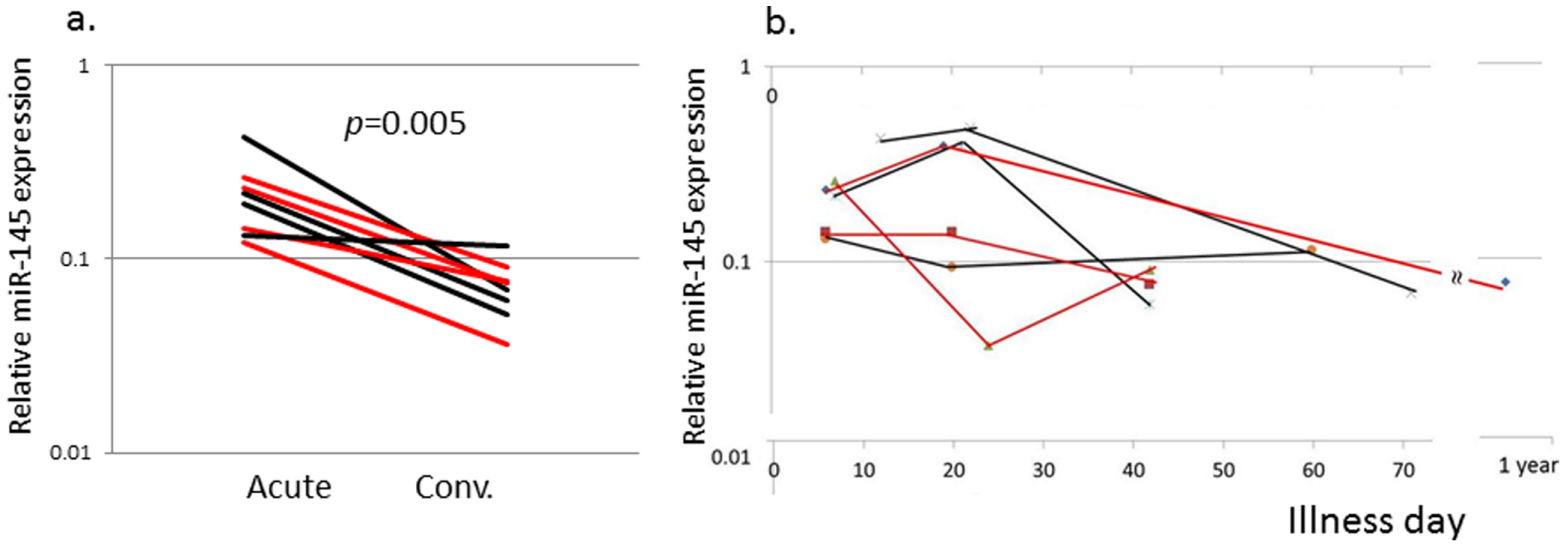
miR-145 transcript levels in subjects with or without coronary artery aneurysms. RT-PCR was performed on an independent cohort of 4 subjects with coronary artery aneurysms and 4 subjects with normal coronary artery internal dimensions measured by echocardiography. a. Relative miR-145 levels for paired samples from acute and convalescent samples. b. Relative miR-145 levels by Illness day of sample collection. Red line: subjects with coronary artery aneurysm, Black line: subjects with normal coronary arteries.

Levels of the six differentially expressed miRs with an adjusted p-value <0.001 and two miRs of biologic interest (miR-23a and miR-145*) were measured in an independent KD cohort (Table 1) using qRT-PCR with stem-loop RT-primers designed for the dominant sequences (Table S4). Differential expression comparing acute vs. convalescent samples was validated for six of the eight miRs (miR-143, -199b-5p, -618, -223, -145 and miR-145*) (Figure 2). The acute levels of the eight miRs analyzed by qRT-PCR did not correlate with Z_worst_ (data not shown). The correlation between miR-145 expression levels and coronary artery aneurysm formation was further tested in an independent cohort of 4 subjects with coronary artery aneurysms and 4 subjects with normal coronary artery internal dimension by echocardiography (Figure 3). The increase in miR-145 levels was a universal response during the acute phase of the vasculitis, regardless of the presence or absence of aneurysms.

### miRs Differentially Expressed in Acute KD vs. Adenovirus Infection

**Figure 4:**
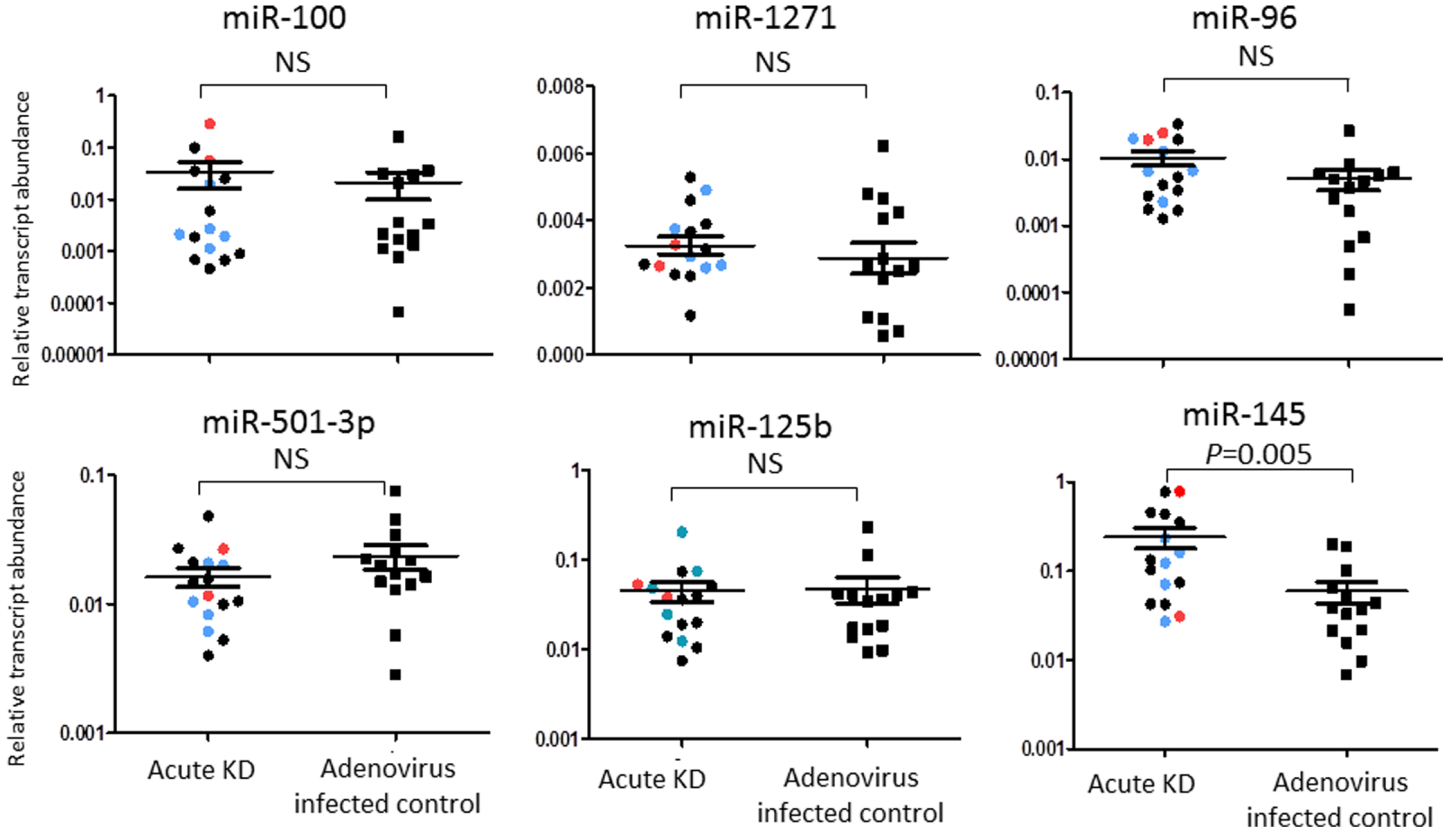
qRT-PCR validation of miRs differentially expressed in whole blood samples from acute KD and adenovirus-infected patients. qRT-PCR confirmed differential expression of miR-145 in an independent cohort of 16 acute KD subjects and 14 acute adenovirus-infected controls. Red dot: subjects with coronary artery aneurysm; Blue dot: subjects with dilated coronary arteries; Black dot: subjects with normal coronary arteries. The Mann Whitney test was used to obtain *p*-values. NS = not significant.

To define an acute KD-specific miR profile, we compared miR reads from acute KD samples with those from children with acute adenovirus infection. In adenovirus infected control subjects (n = 6), 590 miRs were expressed with an absolute expression of 1 count in at least 1 sample. Eight miRs were differentially expressed between KD and adenovirus blood samples, and all were lower in adenovirus infection (Table 3). Among these, only miR-145 was confirmed to also be differentially expressed between acute and convalescent KD samples (Figure 2). Four miRs (miR-100, -1271, -96, and -501-3p) with a p-value <0.001 and miRs with biological interest (miR-145, miR-125b) were tested by qRT-PCR in independent KD and adenovirus samples (Table S1) using stem-loop RT-primers designed for the dominant sequences (Table S5). Differential expression between the groups was confirmed only for miR-145. (Figure 4).

**Table 3:** Differentially expressed miRs: Acute KD vs. Adenovirus-infected control subjects.

	Expression levels†			
miR	Acute KD	Control	Fold difference	*p*-value‡
miR-100	1.58	0.18	8.6	2.60E-09
miR-1271	0.024	0.00086	27.4	1.05E-08
miR-96	0.46	0.092	5.0	8.99E-06
miR-501-3p	0.43	0.082	5.2	4.84E-05
miR-125b	0.20	0.052	3.8	3.99E-03
miR-127-3p	0.46	0.16	2.9	1.04E-02
miR-99b	0.82	0.28	3.0	3.54E-02
miR-145	0.067	0.025	2.6	4.07E-02

† read counts normalized by total reads per sample.

‡ corrected for multiple sample comparison, shown miRs with adjusted *p*-value ≤0.05.

### Correlation between miR Levels and Peripheral Blood Cell Populations

**Figure 5:**
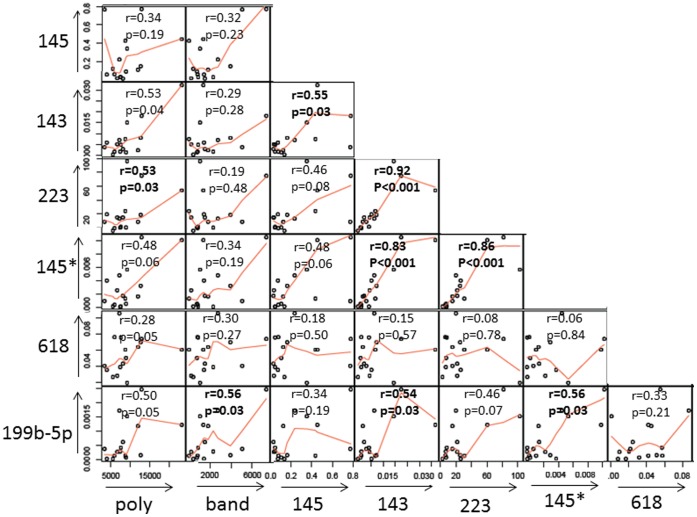
Correlation between miR levels and absolute neutrophil and band counts. Relative miR levels from acute KD whole blood were plotted against each other and against absolute neutrophil and band counts measured in the same blood sample.

Dynamic changes in cell populations circulating during the acute and convalescent stages of KD have been well-described [24]. Cell type-specific expression of miR-143/145 and miR-223 has been reported [25], [26]. To determine whether differential expression of miRs simply reflected changes in peripheral blood cell populations, six miRs (miR-143, -145, -199b-5p, 223, -618, -145*) that showed differential expression in acute vs. convalescent KD blood samples were compared with absolute cell counts for immature and mature neutrophils, eosinophils, monocytes, lymphocytes, and platelets during the acute stage of KD. miR-143, -145*, -223 and -199b-5p showed weak correlation with the absolute neutrophil count, (r = 0.53, *p* = 0.04 for miR-143, r = 0.48, *p* = 0.06 for miR-145*, r = 0.53, *p* = 0.03 for miR-223 and r = 0.50, *p* = 0.05 for miR-199b-5p) (Figure 5). However, miR-145 showed no correlation with absolute neutrophil count (r = 0.34, *p* = 0.19) or total white blood cell count (r = 0.42, P = 0.1). miR-199b-5p showed weak correlation with the band neutrophil count, (r = 0.56, *p* = 0.03). No other significant correlations were noted between other miRs and any cell population (data not shown). This suggests that the differences in these miR expression levels could not be simply explained by differences in circulating cell numbers. Analysis of correlations between miRs showed high correlation among three miRs (miR-143 and 145*, r = 0.83, miR-143 and miR-223, r = 0.92, miR-145* and miR-223, r = 0.86) but weaker correlations between miR-145 and those three miRs (miR-143, r = 0.55, miR-145*, r = 0.48 and miR-223, r = 0.46) (Figure 5).

### Extracellular Vesicles in Acute KD

**Figure 6:**
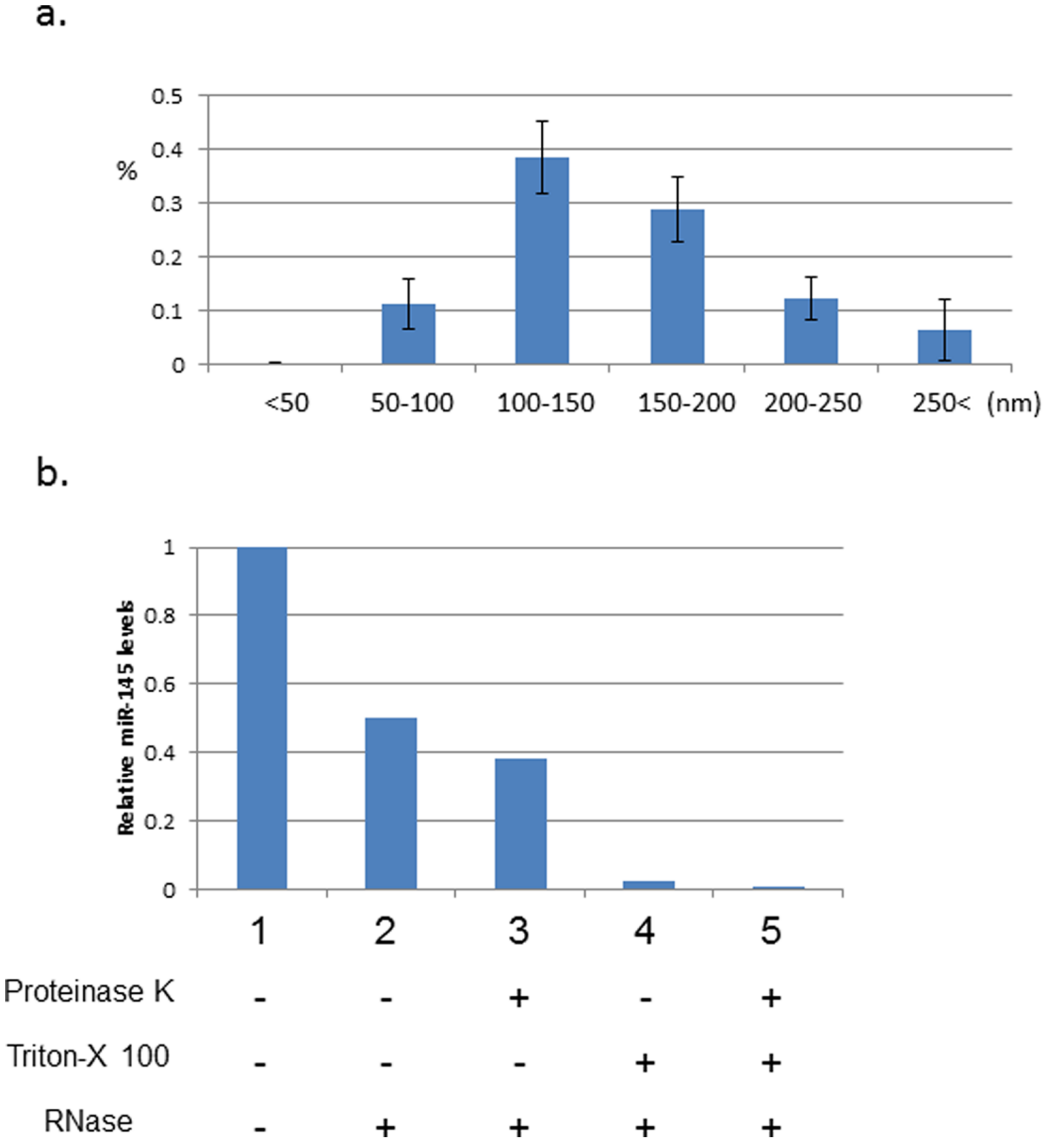
Extracelluar vesicles isolated from the plasma of acute KD subjects. a. Size distribution of extracellular vesicles from plasma. b. miR-145 analysis in extracellular vesicles. Vesicles isolated from whole blood from 14 acute KD subjects were pooled and divided into 5 treatment groups. qRT-PCR levels were compared to the “no treatment” sample.

miR-145 is highly expressed in vascular smooth muscle cell (VSMC) where it mediates phenotype switching [27]. Transfer of miR-145 in small extracellular vesicles between endothelial cells and smooth muscle cells has been recently reported [28]. To test the hypothesis that miR-145 circulates in extracellular vesicles in acute KD subjects, we isolated small extracellular vesicles from plasma (Figure S2a) and analyzed them for miR-145 content. Extracellular vesicles of approximately 100 nm in diameter were observed by transmission electron microscopy (TEM) in the resuspended pellets (Figure S2b.). CD63, a tetraspanin strongly enriched in extracellular vesicles such as exosomes [29], was also detected by Western blotting of a suspension of vesicles (Figure S2c.). The size distribution of vesicles isolated from 14 acute KD subjects (Table 1, Table S1) were 50–200 nm in ≥90% of vesicles, with 11% of the vesicles in the range of 50–100 nm (Figure 6a).

To determine if miR-145 circulates within extracellular vesicles with a phospholipid membrane in the plasma of KD patients during acute phase, we isolated and pooled vesicles from the plasma of 14 acute KD subjects and divided the pool into 5 treatment groups [13]. Plasma samples were pooled to maximize the yield of vesicles, although this precluded our making assessments of individual patients. Treatment with Triton X-100 to disrupt the phospholipid membrane before RNase treatment led to degradation of 40% of the miR-145 transcripts (Figure 6b, column 3 vs. 5), suggesting almost half of the miR-145 in plasma is associated with phospholipid-bound, extracellular vesicles.

### Target Prediction and Pathway Enrichment Analysis for miRs

To predict functions of the differentially expressed miRs, we identified pathways in which the miR target genes participate. The top associated pathway was the TGF-β signaling pathway (*p* = 1.4×10^−5^) with 17 genes targeted by six miRs whose dynamic transcript changes between acute and convalescent samples were validated by RT-PCR (Figure S3).

**Figure 7:**
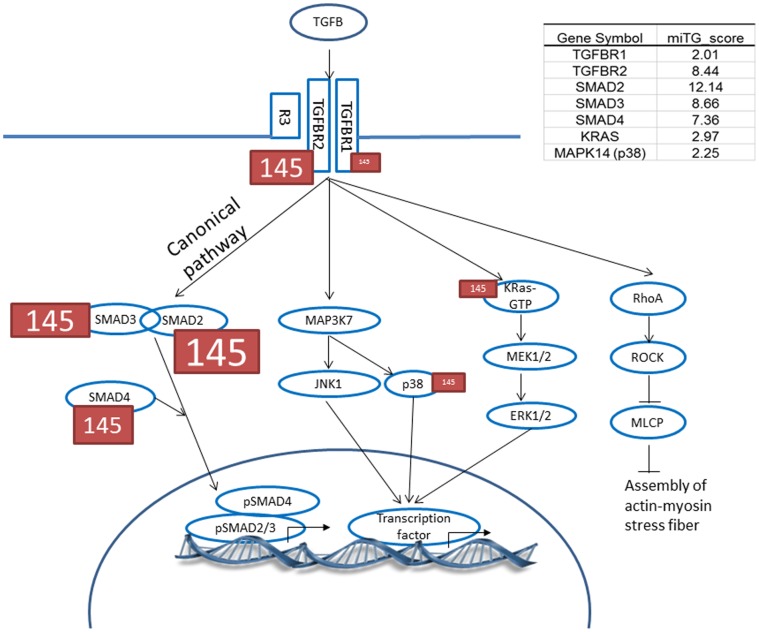
Predicted miR-145 targets in the TGF-β pathway. Higher miTG score is illustrated by larger font. The miTG score is a prediction score based on binding behavior, which includes free energy calculations, between miR driver sequence in the 5′ and 3′UTR sequences of the target gene and the degree of conservation in other species.

TGF-β signaling activates the canonical (SMAD-dependent) signaling cascade and non-canonical cascades such as the mitogen-activated protein kinase (MAPK), ERK, and Rho pathways. To study if differentially expressed miRs preferentially influenced the canonical vs. the non-canonical signaling pathway, we searched predicted targets for miR-145 in these pathways (Figure 7). Although predicted targets were observed in both canonical and non-canonical cascades, SMAD2, 3, and 4, all three key canonical cascade signaling molecules were targeted by miR-145 with higher miTGscores (12.14, 8.66 and 7.36, respectively) compared to the MAPK non-canonical pathway (2.97 and 2.25 for K-ras and p38, respectively) (Figure 7, Table S6).

## Discussion

Using miR sequencing of KD acute and convalescent whole blood samples as a discovery approach, we identified 6 differentially expressed miRs (miRs-143, -199b-5p, -618, -223, -145 and -145*) that were validated in an independent cohort by qRT-PCR. Among these, miR-145 was uniquely expressed in acute KD vs. acute adenovirus infection. Importantly, the level of miR-145 did not significantly correlate with peripheral white blood cell counts, suggesting that this miR mediates biologic processes distinct from processes associated with elevation of these cell counts. Predicted targets of miR-145 are highly enriched for genes that play critical roles in the TGF-β signaling pathway.

### Role of miR-143/145 in Vascular Biology

miR-143 and -145 are encoded in close proximity to each other on human chromosome 5 (≈1.7Kb apart) and share critical roles in the differentiation of neutrophils [30], stem cells [31], [32], and VSMC [27], [31], [33]–[35]. In acute KD samples, levels of miR-143 and miR-145*, but not levels of miR-145, correlated with mature neutrophil counts. In the vasculitis of KD, neutrophils participate in the early destruction of the media and are detected in the damaged coronary arterial wall from autopsies of KD patients who died within 14 days after fever onset [8], [36]. Although miR-143/145 is usually considered to be a bicistronic locus under the control of a common promoter [33], the existence of an internal regulator for miR-145 transcription has been suggested in stem cells and could be operating in other cell types [34]. Levels of miR-143 and miR-145* were highly correlated with each other but showed only weak correlation with miR-145 (Figure 5).

Recently researchers have focused on the importance of miR-143/145 in VSMC phenotype switching [27], [31], [33]–[35]. LacZ reporter gene experiments have shown that miR-143/145 is initially expressed in the developing murine heart at an early embryonic stage (E8.5 to E9.5) and that the expression becomes restricted to SMCs of various organs including the aorta, heart, and coronary arteries during subsequent fetal stages (E16.5) [31], [33], [35]. VSMC, unlike cardiomyocytes and skeletal muscle cells, retain plasticity and can switch from an α-SMA expressing contractile phenotype (regulated by miR-145) to a proliferative, synthetic phenotype [27], [37], [38]. α-SMA positive/smoothelin negative myofibroblasts, whose generation is influenced by TGF-β, participate in the destruction of the arterial wall in acute KD and it is intriguing to speculate that miR-145 modulates the generation of myofibroblasts from VSMC [9]. Another feature of the vascular injury in acute and subacute KD is the development of myointimal proliferation, which is known to be regulated by miR-145 [27], [33]–[35].

Several lines of evidence suggest that animal models of non-inflammatory vascular injury or human diseases characterized by chronic vascular inflammation are associated with low levels of miR-145. miR-145 expression was down-regulated in tissues at the site of experimental injury-induced lesions in animal models [31], [34], [39] and in human aortic aneurysms [39]. Similarly, levels of miR-145 in the plasma or serum of patients with stable coronary artery disease (CAD) were low compared to healthy controls [40]. Thus, conditions associated with chronic vascsular inflammation are associated with low levels of miR-145. In contrast, levels were high in our acute KD subjects and lower in the convalescent phase when inflammation had resolved. We postulate that miR-145 down-regulates TGF-β signaling, thus leading to resolution of the myofibroblast-mediated acute inflammation.

Although miR-145 is predicted to regulate transcript levels of several genes in the TGF-β pathway, including SMAD2, 3 and 4 (Table S6), confirmatory luciferase reporter gene experiments have not yet been performed. Studies of the target genes of miR-145 may deepen our understanding of the role of miR-145 in KD pathogenesis. miR-145 also affects VSMC phenotype by targeting calcium/calmodulin-dependent protein kinase II, delta (Camk2d) [31] and angiotensin converting enzyme (Ace) [33]. Genetic variation in CAMK2D [41] and ACE [42]–[45] has been linked to KD susceptibility and outcome.

miRs can be transported from cell to cell by small (∼100 nm) extracellular vesicles and can block mRNA translation in recipient cells [46]. Thus, miRs carried by extracellular vesicles may play a significant role in disease pathogenesis [47]. Communication between endothelial cells and VSMCs by extracellular vesicles carrying miR-143/145 has been reported [13]. We found that approximately 40% of miR-145 in plasma was contained within small extracellular vesicles (Figure 6). Identifying the cellular origins and targets of these vesicles may contribute to our understanding of the role of miR-145 in KD. The use of circulating miRs and miRs in extracellular vesicles as biomarkers has been pursued for cancer and cardiovascular disease [47], [48]. Future studies with expanded cohorts of KD and febrile control patients will explore whether miR-145 contained within extracellular vesicles may serve as a biomarker for acute KD.

We identified three additional miRs that were differentially expressed in whole blood between the acute and convalescent stages of KD and were validated in independent patient cohorts. miR-199b is regulated by the transcription factor NFAT, which has been implicated in KD pathogenesis [5].This miR is increased in the setting of heart failure in humans [49]. miR-23a ,which is also regulated by NFAT and up-regulated during cardiac hypertrophy [50], was differentially expressed based on the sequencing data (Table 2), but was not confirmed by qRT-PCR in the independent cohort (Figure 2). While clinically significant ventricular dysfunction is rare in acute KD, it is well-documented that all KD patients have some degree of myocardial inflammation during the acute phase [51]. In our analysis, it is interesting to note that the subject with highest miR-199b-5p levels by qRT-PCR had a low ejection fraction (56%) during the acute phase, suggesting more severe myocardial inflammation (data not shown).

miR-223 was also significantly differentially expressed between acute and convalescent samples. This miR is reported to be expressed in neutrophils, eosinophils, monocytes and platelets [25], [26]. miR-223 acts to fine-tune granulocyte production by the bone marrow and the inflammatory response by modulating the proliferation of granulocyte progenitors and maturation of neutrophils [52]. In our study there was weak correlation between miR-223 expression and absolute neutrophil count (r = 0.53) but not with counts of eosinophils, monocytes, or platelets. miR-223 expression levels showed strong correlation with miR-143 and miR-145*, suggesting that these miRs may be regulated by a shared mechanism (Figure 5).

miR-618, another differentially expressed miR, has a polymorphism (rs2682818 at Chr. 12q21.31) on the complementary strand in the seed region [53]. However, the mature sequence is not affected by this polymorphism and the functional significance is unknown. None of the KD genetic studies published to date has identified an association between KD and this locus on Chr. 12.

### Pathway/target Genes

Pathway analysis of the predicted target genes for the six differentially expressed miRs identified the TGF-β signaling pathway. Although most miRs down-regulate translation of their target genes, enhanced gene expression regulated by miRs has been reported [52]. In zebrafish, miRs play an important role in stabilizing intracellular processes by buffering expression fluctuations of opposing genes in the TGF-β pathway [10]. It is intriguing to speculate that the abundance of miR targets in the SMAD 2, 3, 4 canonical pathway might lead to reduced signaling and to preferential shunting down the non-canonical MAPK pathway that has been recently implicated in other aneurysm syndromes [54]. Experiments by Long and Miano using human coronary artery smooth muscle cells demonstrated the time-dependent increase in miR-145 levels and increase in non-canonical TGF-β pathway molecules, pERK and pp38MAPK, by Western blotting, following TGF-β stimulation [38]. In addition, work by Zhu et al. showed increased levels of miR-145 and decreased levels of SMAD3 following TGF-β stimulation of human fibrobalsts [55]. In KD we have demonstrated increased signaling through the canonical TGF-β pathway in the coronary artery wall [9]. In a discovery analysis of the miR-ome in acute and convalescent KD, we discovered increased levels of miR-145 and other miRs that also negatively regulate TGF-β signaling (Figure 7, Figure S3). We postulate that miR-145 acts as a negative regulator of TGF-β signaling through the canonical pathway and thus may contribute to the down regulation of inflammation in the arterial wall.

Previously, we demonstrated that transcripts for TGFBR3 and SMAD3 were reduced during acute KD by microarray analysis and reduced expression of TGFBR3 was confirmed by qRT-PCR [7]. Although these transcripts are predicted to be targets of miR-223, -618, -143 and -145, we did not see a correlation between these miRs and transcript levels of *TGFBR3* or *SMAD3* in whole blood from acute KD patients (data not shown). It may be that analysis of changes in the peripheral blood does not necessarily reflect the status in the arterial wall, where these miRs may be acting to negatively regulate these molecules.

### Strength and Limitation

We recognize several strengths and limitations to our study. This is the first study to analyze miR sequences in KD. The novel findings of differentially expressed miRs enriched for the TGF-β pathway further emphasizes the important of this pathway in KD pathogenesis. This is also the first study to examine extra-cellular vesicles in acute KD. The study demonstrated that miRs mediating KD pathogenesis may be associated with extracellular vesicles. The role of these vesicles in inter-cellular communication is a new intriguing avenue of research. The small sample size is a limitation in this type of study and may impact its general applicability. However, sequencing results were validated using a different method in two independent cohorts of KD and adenovirus-infected subjects. Another limitation is the use of RNA extracted from whole blood, which contains diverse cell types, including smooth muscle progenitor cells, extracellular vesicles and Argonaut protein-complexed miRs [56]. Therefore the cellular source of the differentially expressed miRs is unclear. We did not see any correlation between aneurysm formation and miR-145 levels. This may be related to sampling in the peripheral blood vs. in the arterial wall.

In summary, our study demonstrates that elevated miR-145 levels are found in whole blood from patients with acute KD. To the extent that many of the predicted targets of miR-145 are involved in TGF-β signaling, we propose that miR-145 downregulation of the canonical TGF-β pathway may have an important role in recovery from the acute vasculitis. These results lend further weight to the importance of the TGF-β signaling pathway in acute KD.

## Supporting Information

Figure S1 **Aligned isomiRs.** Comparison of reads from representative acute KD samples (subject 2 for miR-143 and subject 4 for miR-145) aligned with deposited sequences of miR-143 and miR-145 in miRBase. Underlined red sequences = miRBase submitted; boxed sequences = dominant sequence (most reads for that miR).
(TIF)

Figure S2 **Extracellular particles isolation.** a. Flowchart of isolation of extracellular particles. b. Transmission electron microscopic (TEM) analysis of extracellular particles. Small particles with size of 50 nm-200 nm were observed by TEM in the resuspended pellets isolated by ultracentrifugation. Three µl of extracellular particles were fixed in 2% paraformaldehide, stained with uranyl acetate, stacked on a grid, and examined in a Joel 1200 EXII transmission electron microscope (TEM) (UCSD CMM EM Facility). c. Western blot of CD63 expression in resuspended pellets isolated by ultracentrifugation. Vesicle samples were lysed in cell lysis buffer (300 mM NaCl 50 mM Tris, pH 7.4 (pH of Tris base adjusted with 6 N HCl) 0.5% Triton X-100 with cOmplete antiprotease cocktail (Roche)). 7.5–30 µg of protein were separated on a 12% polyacrylamide gel (SDS-PAGE). For Western blotting, proteins were transferred from polyacrylamide gels to polyvinylidene fluoride membrane (BioRad, Hercules, CA). Membranes were blocked in TBS containing 5% (w/v) non-fat dry milk and 0.1% (w/v) Tween 20 and probed with mouse monoclonal anti-CD63 (ab8219, abcam). Primary antibodies were probed with horseradish peroxidase-labeled anti-mouse antibodies (NA931VS, GE Healthcare) and detected using the Enhanced Chemiluminescence Detection kit (ECL plus Western blotting detection system Cat. RPN2131, GE Healthcare Life Sciences).
(TIF)

Figure S3 **miR target genes are enriched for genes in TGF-β pathway.** Target genes predicted to be regulated by the six differentially expressed miRs are in red font.
(TIF)

Table S1 **Characteristics of subjects.**
(TIF)

Table S2 **Sequenced small RNA species.**
(TIF)

Table S3 **Differentially expressed all miRs: Acute vs. Convalescent KD.**
(TIF)

Table S4 **1^st^ and 2^nd^ dominant sequences and reads for each subjects of differentially expressed miRs between Acute KD and convalescent.**
(TIF)

Table S5 **1^st^ and 2^nd^ dominant sequences and reads for each subjects of differentially expressed miRs between Acute KD and adenovirus infected control.**
(TIF)

Table S6 **Predicted targets of TGF-β pathway.**
(TIF)
