# Supplementary figures and images for: Differential Expression of miR-145 in Children with Kawasaki Disease
Source: PLoS One. 2013 Mar 6;8(3):e58159. doi: 10.1371/journal.pone.0058159 (PMC3590129; doi:10.1371/journal.pone.0058159)

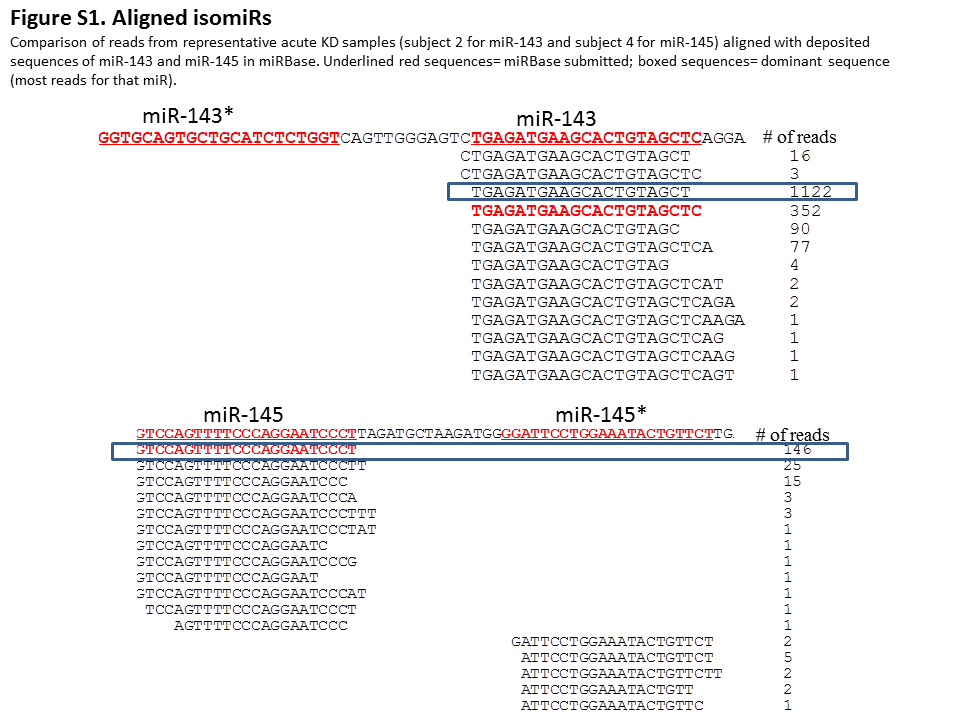

Supplement: Figure S1 — Aligned isomiRs. Comparison of reads from representative acute KD samples (subject 2 for miR-143 and subject 4 for miR-145) aligned with deposited sequences of miR-143 and miR-145 in miRBase. Underlined red sequences = miRBase submitted; boxed sequences = dominant sequence (most reads for that miR). (TIF) [file pone.0058159.s001.tif]

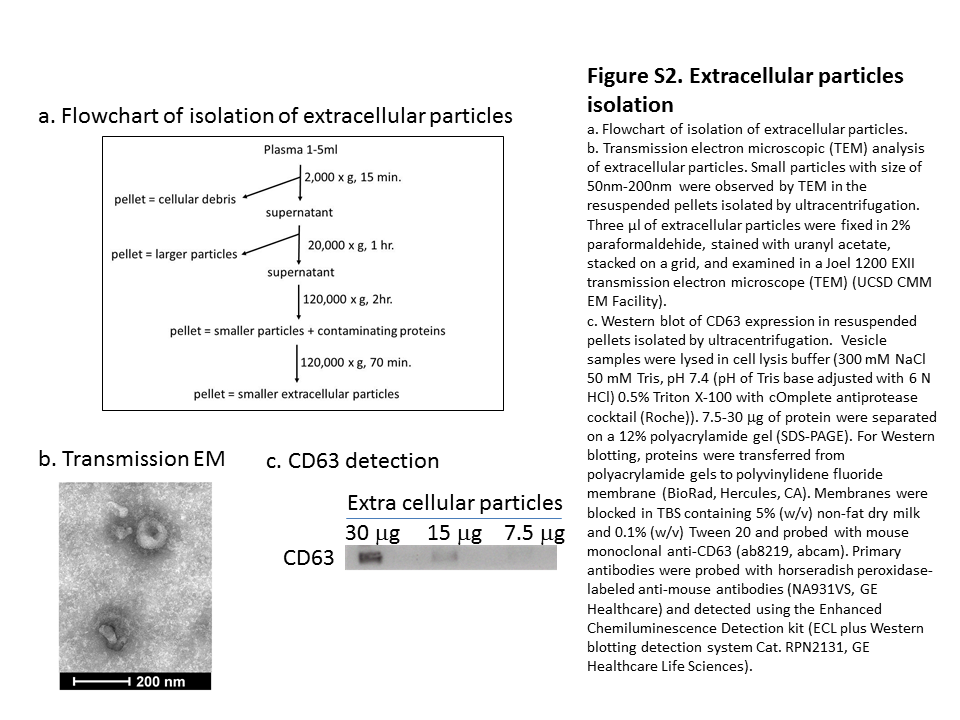

Supplement: Figure S2 — Extracellular particles isolation. a. Flowchart of isolation of extracellular particles. b. Transmission electron microscopic (TEM) analysis of extracellular particles. Small particles with size of 50 nm-200 nm were observed by TEM in the resuspended pellets isolated by ultracentrifugation. Three µl of extracellular particles were fixed in 2% paraformaldehide, stained with uranyl acetate, stacked on a grid, and examined in a Joel 1200 EXII transmission electron microscope (TEM) (UCSD CMM EM Facility). c. Western blot of CD63 expression in resuspended pellets isolated by ultracentrifugation. Vesicle samples were lysed in cell lysis buffer (300 mM NaCl 50 mM Tris, pH 7.4 (pH of Tris base adjusted with 6 N HCl) 0.5% Triton X-100 with cOmplete antiprotease cocktail (Roche)). 7.5–30 µg of protein were separated on a 12% polyacrylamide gel (SDS-PAGE). For Western blotting, proteins were transferred from polyacrylamide gels to polyvinylidene fluoride membrane (BioRad, Hercules, CA). Membranes were blocked in TBS containing 5% (w/v) non-fat dry milk and 0.1% (w/v) Tween 20 and probed with mouse monoclonal anti-CD63 (ab8219, abcam). Primary antibodies were probed with horseradish peroxidase-labeled anti-mouse antibodies (NA931VS, GE Healthcare) and detected using the Enhanced Chemiluminescence Detection kit (ECL plus Western blotting detection system Cat. RPN2131, GE Healthcare Life Sciences). (TIF) [file pone.0058159.s002.tif]

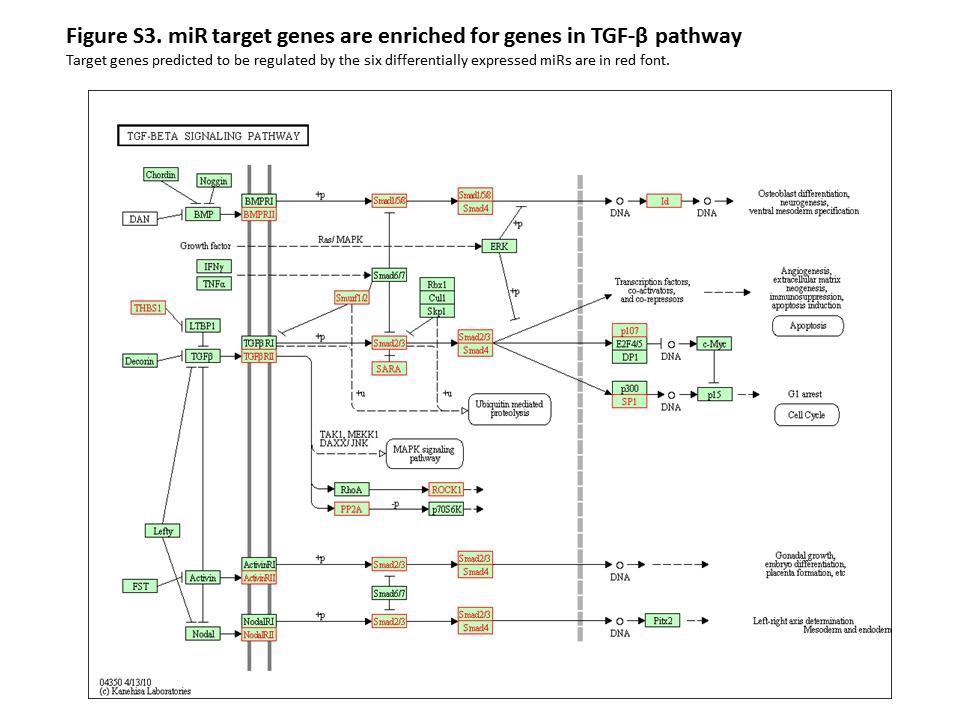

Supplement: Figure S3 — miR target genes are enriched for genes in TGF-β pathway. Target genes predicted to be regulated by the six differentially expressed miRs are in red font. (TIF) [file pone.0058159.s003.tif]

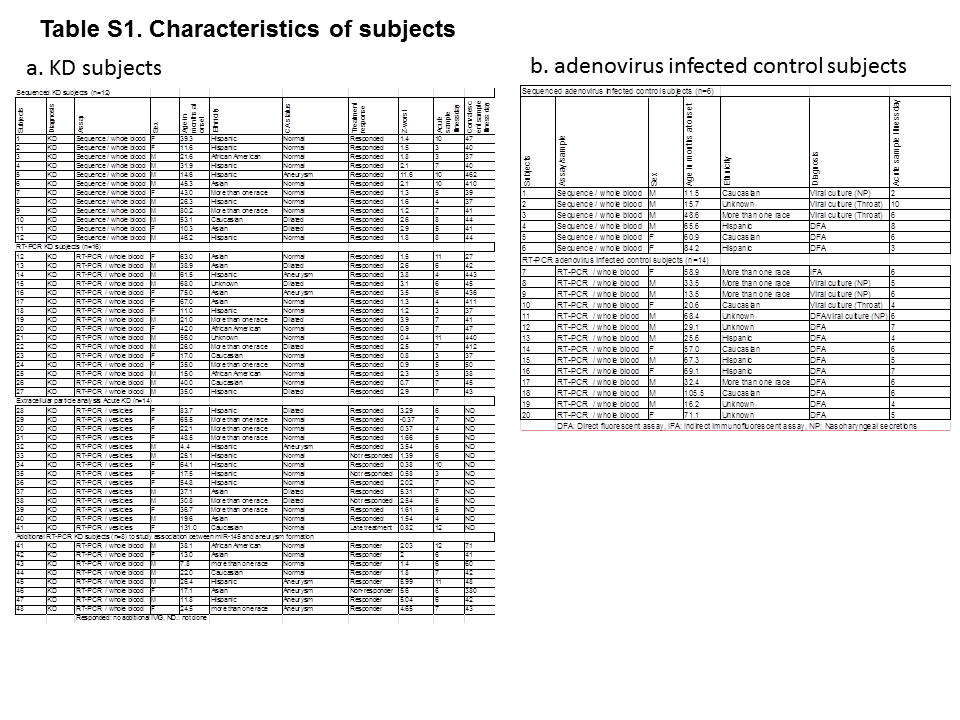

Supplement: Table S1 — Characteristics of subjects. (TIF) [file pone.0058159.s004.tif]

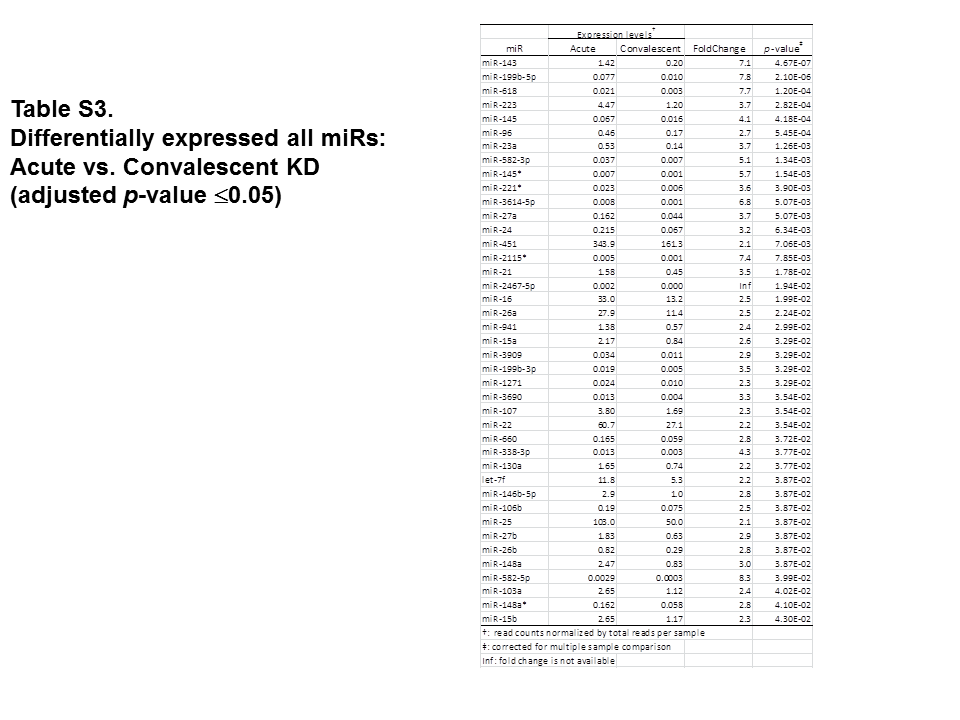

Supplement: Table S3 — Differentially expressed all miRs: Acute vs. Convalescent KD. (TIF) [file pone.0058159.s006.tif]

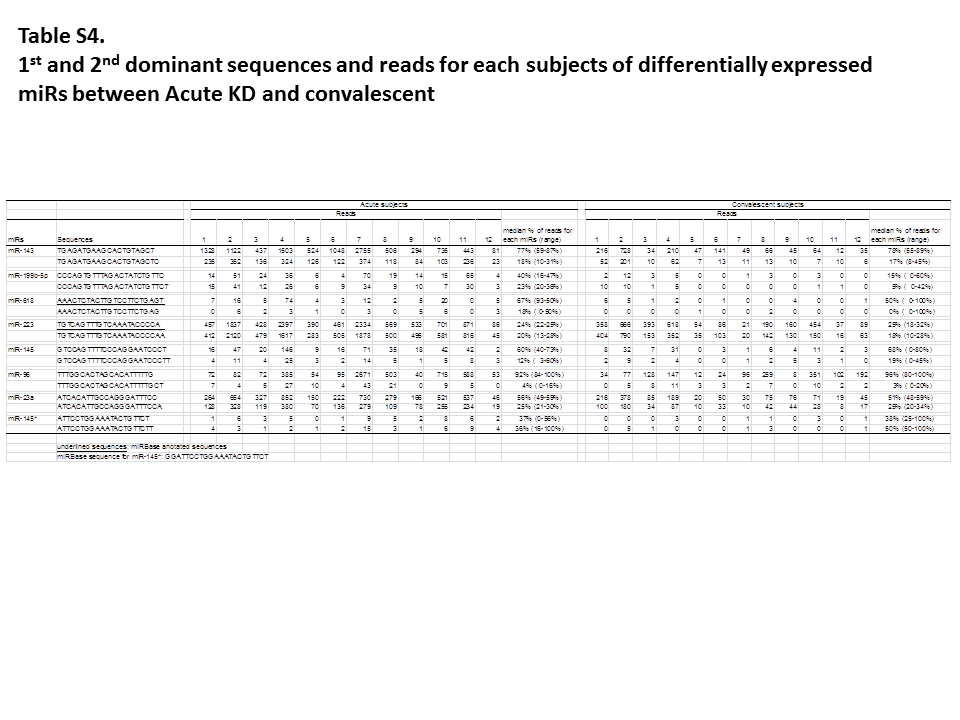

Supplement: Table S4 — 1st and 2nd dominant sequences and reads for each subjects of differentially expressed miRs between Acute KD and convalescent. (TIF) [file pone.0058159.s007.tif]

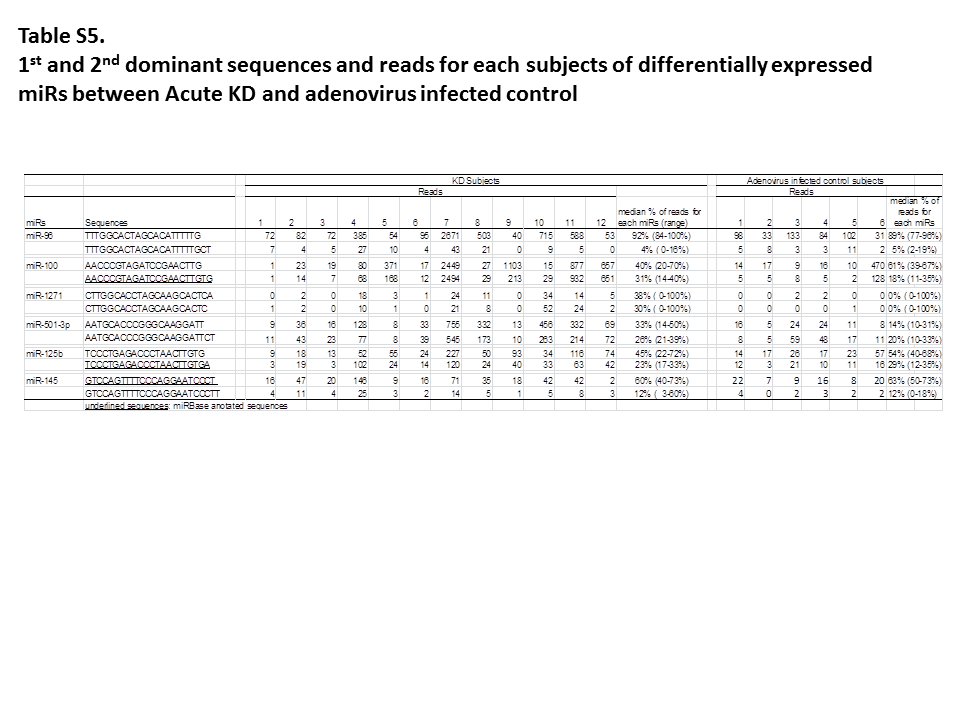

Supplement: Table S5 — 1st and 2nd dominant sequences and reads for each subjects of differentially expressed miRs between Acute KD and adenovirus infected control. (TIF) [file pone.0058159.s008.tif]
